# Systems Biology and Cytokines Potential Role in Lung Cancer Immunotherapy Targeting Autophagic Axis

**DOI:** 10.3390/biomedicines11102706

**Published:** 2023-10-05

**Authors:** Riya Khilwani, Shailza Singh

**Affiliations:** Systems Medicine Laboratory, National Centre for Cell Science, SPPU Campus, Ganeshkhind Road, Pune 411007, India; riyakhilwani@nccs.res.in

**Keywords:** cytokines, autophagy, NSCLC, therapeutics, IL6, IL17, IL23

## Abstract

Lung cancer accounts for the highest number of deaths among men and women worldwide. Although extensive therapies, either alone or in conjunction with some specific drugs, continue to be the principal regimen for evolving lung cancer, significant improvements are still needed to understand the inherent biology behind progressive inflammation and its detection. Unfortunately, despite every advancement in its treatment, lung cancer patients display different growth mechanisms and continue to die at significant rates. Autophagy, which is a physiological defense mechanism, serves to meet the energy demands of nutrient-deprived cancer cells and sustain the tumor cells under stressed conditions. In contrast, autophagy is believed to play a dual role during different stages of tumorigenesis. During early stages, it acts as a tumor suppressor, degrading oncogenic proteins; however, during later stages, autophagy supports tumor cell survival by minimizing stress in the tumor microenvironment. The pivotal role of the IL6-IL17-IL23 signaling axis has been observed to trigger autophagic events in lung cancer patients. Since the obvious roles of autophagy are a result of different immune signaling cascades, systems biology can be an effective tool to understand these interconnections and enhance cancer treatment and immunotherapy. In this review, we focus on how systems biology can be exploited to target autophagic processes that resolve inflammatory responses and contribute to better treatment in carcinogenesis.

## 1. Introduction

**Figure 1:**
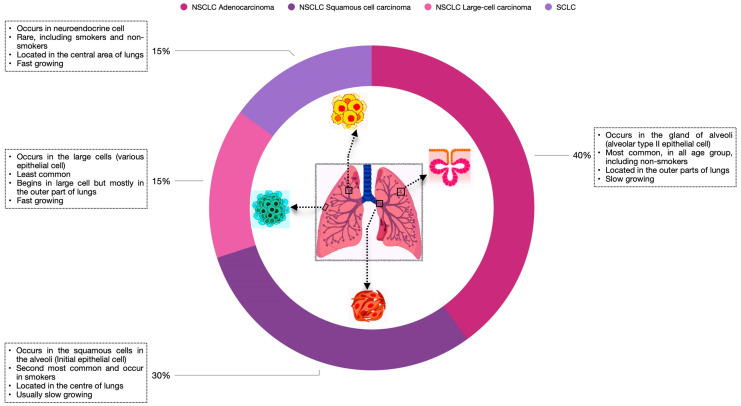
**Overview of lung cancer and its types.** Lung cancers are classified into two types: SCLC and NSCLC. NSCLC includes three major subtypes: adenocarcinoma, squamous cell carcinoma, and large-cell carcinoma. The figure shows the location where tumor development takes place. Also, the frequency, growth rate, and specific age group are defined for each cancer type.

For many years, lung cancer (LC) has been the most common cause of cancer-associated mortality. Based on histological evaluations, LC is of two types: non-small cell lung cancer and small-cell lung cancer. NSCLC accounts for 85% of the total diagnosed cases, whereas small-cell lung cancer accounts for the remaining 15%. Depending on the location and the cell type where LC occurs, NSCLC is divided into three types: adenocarcinoma (about 40%), squamous cell carcinoma (about 30%), and large-cell carcinoma (about 15%) [1], as illustrated in Figure 1. Although several targeted therapies have revolutionized the treatment of NSCLC, significant improvements are still needed to its diagnosis, staging, and characterization. At present, several cytokines promote inflammatory events in cancer. It has been observed that cytokines play a key role in transducing the signaling mechanisms that modulate inherent autophagic events, leading to disease progression [2,3]. Therefore, to overcome this effect, it is of ultimate importance to identify the principal signaling pathway and its intermediates that can be targeted to achieve a novel therapeutic strategy in lung cancer [4,5].

Cancer is a multifaceted inflammatory disease that displays distinct growth characteristics, including continued proliferation, immune evasion, immortality, angiogenesis, invasion, metastasis, and cell death avoidance [6]. The capability of cancer cells to escape apoptotic mechanisms is a well-known fact that allows for the proliferation and metastasis of tumor cells [7]. In the present report, the accumulation of impaired proteins and non-functional organelles suggests injured autophagic mechanisms and an imbalance in tissue homeostasis, which further indicates the role of autophagy in the development of cancer [8]. The detailed mechanism and crosstalk between cancer and autophagy are less understood [9], but several studies indicate that autophagy is a double-edged sword that has long been connected to both cancer development and immunotherapy. This suggests the role of autophagy as both a tumor promoter and an inhibitor [10]. In fact, other research implies that the autophagy process is regulated by transcription factors, which are transduced by the signaling axis and may have an effect on tumor progression or repression. Therefore, it is important to deduce which signaling mechanism has an inherent role in upregulating autophagic protein expression and which immunomodulatory process exacerbates the biology behind cancer [11].

Different stress stimuli, such as internal and external stress, activate the body’s defense mechanisms, which are essential for maintaining cellular homeostasis [12]. One such mechanism activated by stress is autophagy, wherein protein aggregates, misfolded proteins, and damaged organelles are engulfed in double-membrane vesicles, which then fuse with the lysosome, producing energy via the degradation of subcellular components [13]. In general, autophagy can be classified into selective and non-selective types. Selective autophagy operates by recognizing specific targets, like intracellular pathogens, impaired organelles, and their proteins [14]. However, non-selective autophagy works at the ground level, marking the cytoplasmic content for degradation [15]. In addition to preserving organelle and protein quality, innate autophagy operates at a fundamental level together with ubiquitin-mediated proteasomal degradation in order to ensure the elimination of faulty proteins [16]. It is also described as a pathway to process and present the antigen to T-cells. This reveals the importance of autophagy in host defense, which is also associated with the generation of immune responses in inflammatory diseases like cancer [17].

Understanding the cancer regulatory network in mammalian hosts is difficult due to the complexity of the system. Molecular analysis helps to elucidate the disease pathology at the bio-molecular interactions; however, this level of understanding is insufficient to link the physiological response. Despite the fact that the standard methods are used in biology, the complexity of biological systems presents new opportunities for discovery [18]. In the last ten years, systems biology has become a modern approach to solving such an issue. It is observed as a tool that best correlates genotype to phenotype and studies intricate connections and their emergent features within biological systems. In cancer biology, it strives to provide a more comprehensive understanding of how cancer develops and spreads. Its approaches help to comprehend how the deregulation of signaling events facilitates malignant phenotypes in healthy cells. Although computational methods revolutionized the era of systems biology, various newer approaches have emerged to understand the process behind oncogenesis [19]. A mathematical model is great for understanding the intrinsic behavior and the extracellular crosstalk in the tumor microenvironment. It has also paved the way to grasp the underlying cause of cancer drug resistance to unravel mechanisms to discover potential regimens against cancer [20]. In this review, we detail the autophagy process and how it aids in tumor development, metastasis, and contributes to drug resistance in cancer. Further, we highlight the crucial role of systems biology in modulating the effect generated as a result of physiological defense towards cancer immunotherapy. Finally, we discuss the autophagic process as a therapeutic target for resolving cancer-mediated inflammation.

## 2. Mechanism of Autophagy

Yoshinori Ohsumi received the Nobel Prize in Physiology or Medicine in 2016 for his research on autophagy and its impact on chronic inflammation and diseases [21]. Autophagy, which is derived from the Greek words “auto”, meaning self, and “phage”, meaning eating [22], is a defense mechanism conserved in eukaryotes, including yeasts, flies, plants, and mammals [23], and is responsible for the degradation of molecules enclosed in a double membranous structure to achieve cellular homeostasis. Autophagy is induced by a range of physiological stresses, including oxidative and nutrient stress, non-functional protein aggregates, and organelle dysfunction, and is broadly classified into three major types depending on the delivery of cargo either through lysosomes or vacuoles: microautophagy, macroautophagy, and chaperone-mediated autophagy. Microautophagy allows the lysosomal membrane itself to engulf molecules for degradation. The process is divided into different types depending on the source and the substrate used for degradation: micromitophagy (for mitochondria), micropexophagy (for peroxisomes), and microlipophagy (for lipid droplets). However, CMA uses the HSC70 to identify cargo that has a pentapeptide motif, KFERQ, and allow its docking to LAMP2A. Unlike microautophagy and CMA, macroautophagy refers to an exact autophagy mechanism that is intricately linked to conserved autophagy-related genes for the formation of autophagosomes [24,25,26]. The process of macroautophagy is divided into five different stages: induction, nucleation, membrane maturation, autophagosome-lysosome fusion, and degradation and recycling of products. The stage-by-stage dissection is crucial since each stage contains a specific collection of possible treatment targets for preventing autophagy in humans. In addition, current research raises the possibility that the biological consequences of tumor cell behavior may vary depending on which stage of the autophagy pathway is inhibited [27].

**Figure 2:**
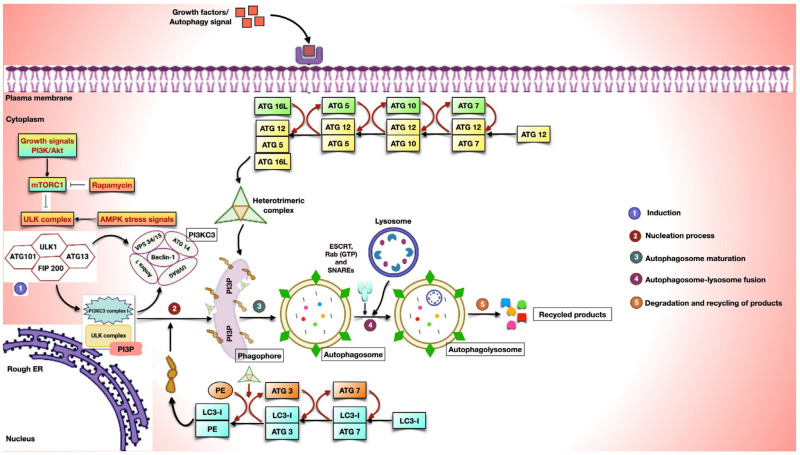
**The complete mechanism of autophagy.** The pathway starts with the autophagy signal that induces the molecular sensor, AMPK, to inhibit its target, mTOR. AMPK activates the ULK complex to initiate the physiological cell death process. This is followed by the formation of the PI3KC3 complex, which unites with the ULK complex to form an isolation membrane. During the second step, the membrane interacts with two different conjugation complexes to facilitate autophagosome elongation and maturation. Once the autophagosome matures, it is ready to fuse with lysosomes for the degradation of products and recycling of nutrients back into the cytosol.

A number of proteins regulate the autophagy process. mTOR, a mammalian target of rapamycin, includes two complexes, mTORC1 and mTORC2 [28], each of which demonstrates a unique function and its ability to develop cancer [29,30]. Of these, activated mTOR complex 1 is known for its prime role in suppressing autophagy via phosphorylating ATG proteins. However, under stress conditions, mTOR gets inhibited, exerting a positive effect on autophagy initiation. During the process, AMPK acts as a molecular sensor that controls mTORC1 and activates autophagy [31]. In the yeast system, the initial stages of autophagy and the mechanism of phagophore formation are clearly understood, whereas in mammals, the process has not been well defined. As detailed in the second figure, the activation of the ULK1 complex induces the process of autophagy. Proteins like ULK1, ATG13, FIP200, and ATG101 constitute the ULK complex [32]. Upon activation, it recruits other autophagic proteins like VPS34, Beclin-1, VPS 15, and ATG14L to form the PI3KC3 complex (Figure 2). This induces the synthesis of PI3P, a molecule that is rich in the membrane of the autophagosome [33]. A phagophore is a sac-like double membrane structure that is often referred to as an isolation membrane. It originates from the ER, Golgi complex, mitochondria, and other organelles and eventually develops into an autophagosome [34]. The sealing of the autophagosome is achieved through the members of the ESCRT family, especially VPS and CHMP2A [35]. However, its maturation is assisted through two ubiquitin-like conjugation systems: the ATG12 conjugation system and the ATG8 conjugation system. In the first conjugation event, an E1-like enzyme, ATG7, and an E2-like enzyme, ATG10, conjugate together to expedite the transfer of ATG5 to ATG12. This results in the formation of a heterodimeric complex that associates with ATG16L to achieve the trimeric structure. The complex thus formed facilitates the second conjugation step and gets occupied on the surface of autophagosomes [36]. During the second event, LC3 matures to interact with the autophagosome in the cytosol, which is indispensable for the initial stages of autophagy [37].

As per the amino acid sequence homology, there are three reported mammalian variants of the ATG8 protein. It includes GABARAPs, LC3, and GATE-16 subfamilies. The GABARAP and LC3 subfamilies are both necessary for effective autophagy. The GABARAP families, including GATE-16, function in the maturation of autophagosomes [38]. However, LC3 helps in the elongation of the phagophore, where a frequency of ATG8 controls the size of autophagosomes [39]. In order to make the system homeostatic, the process requires other proteins to achieve LC3II maturation. It is achieved through the cleavage of proLC3 into LC3I by an E1-like enzyme, ATG 4. Next, with the help of other *ATG* genes, LC3I conjugates with PE to produce mature LC3II. These steps mark the elongation of the isolation membrane to form autophagosomes. The formed autophagosomes then fuse with lysosomes through proteins like SNAREs and Rab7 (small GTPases). Finally, the lysosomes ensure the degradation of engulfed autophagic content and restore important macromolecules back into the cytosol. This inherent process of autophagy enables the production of crucial components and enhances cell survival, which are needed during stressful situations [40]. 

## 3. Autophagy: An Anomalous Defense in Cancer

In cancer biology, autophagy is believed to play a paradoxical role in the genesis and proliferation of cancer cells as well as in the suppression and progression of tumors [10]. According to Kisen et al., when elementary levels of autophagy were calculated in normal and transformed cells, the amount of ubiquitinylated products was found to be higher in healthy cells. These findings suggest a direct relationship between decreased autophagy and cancer [41]. In general, autophagy operates at the basal cellular level in order to clear damaged molecules and suppress inflammation. However, autophagy employed at an inadequate level enhances tumor development in nutrient-deprived cells by preventing the breakdown of non-functional proteins [13]. Autophagy during the early stages prevents the development of tumors. However, after tumor development and malignant transformation, cancer cells have shown increased levels of autophagy, exhibiting resistance to stressful conditions [42].

It is notable that different tumor origins display distinct levels of autophagy; lung cancer has gained resistance and shown high levels of autophagy even in unstressed conditions. Furthermore, the extent of autophagy determines the fate of tumor cells, which is dependent on the origin and type of the tumor, tumor stage, genomic content, and tumor microenvironment [39,43]. Interestingly, it has been reported that the expression level of many tumor suppressor genes and oncogenes influences the autophagic process, where the loss of their regulatory control leads to malignancy [44]. Because of the role of autophagy in regulating genomic stability and preventing tumor formation at early stages, as well as safeguarding cancer cells against various stresses at later stage [45], and also due to its dual role in cancer treatment, autophagy is often referred to as a “double-edged sword” [46].

### 3.1. The Role of Autophagy in Tumor Inhibition

As discussed earlier, autophagy is a conserved process operating at the basal level to get rid of damaged cellular components so as to attain equilibrium. It is the best-known mechanism that limits the destruction of cells and maintains the integrity of the genome. Proteins like mTOR and AMPK negatively regulate tumor suppressor genes such as p53, TSC1, PTEN, and TSC2, which lead to autophagy initiation and tumor suppression. On the other hand, oncogenes including mTOR, Akt, and Bcl-2 inhibit autophagy, suggesting their loss-of-function mutation contributes to autophagic intermediate overexpression [47]. pTEN dephosphorylates PIP3 to PIP2 and modulates the PI3K/Akt/mTOR pathway to promote autophagy. While a mutated copy of PTEN inhibits autophagy in multiple cancers [48]. In addition, the role of p53 in autophagy regulation in cancer is site-dependent. Inside the nucleus, transcription factor p53 stimulates autophagy via TSC1/TSC2 and AMPK activation, while cytoplasmic p53 possesses an inhibitory effect on the autophagic process and induces autophagy only upon its degradation [49,50]. In the majority of cancers, including lung cancer, an alteration in the autophagic pathway is observed as a result of mutations in the PI3K-Akt axis, which are the downstream effectors of important signaling events. Therefore, for autophagy to be functional, it requires control either upstream or downstream of the PI3K pathway [51,52]. Bcl-2, which is an anti-apoptotic protein, interacts with Beclin-1 through its BH3 domain and lowers its affinity for autophagy cell death [53]. In particular, Bcl-2 concentrations are found to be upregulated in almost all cancers [54]. Therefore, we can conclude that the presence of Bcl-2 prevents the onset of autophagy by inhibiting Beclin-1, which suggests the role of Beclin-1 in autophagy.

Various mutation studies acknowledge the importance of the inherent pathway and the important role of autophagic proteins in controlling tumor growth. Recent literature reports the chief role of the *BECNI* gene encoding Beclin-1 as a tumor suppressor, which has been observed by the absence of Beclin-1 in ovarian, prostate, and breast cancer. In vitro and in vivo mouse model studies support the same research findings and suggest the suppression of autophagy and subsequent cell proliferation. These results clearly indicate that Beclin-1 is a potent tumor suppressor that, upon mutation, may negatively regulate autophagy [55]. Moreover, certain studies reflect the decrease in the level of Beclin-1 upon inflammation in cancers like hepatocellular carcinoma and cervical squamous cell carcinoma [56,57]. Additionally, researchers have outlined other autophagy genes as crucial components for cancer control. Proteins such as Bif-1 and UVRAG interact with Beclin-1 and play a major role in promoting autophagy. Like Beclin-1, the loss and depletion of the proteins, respectively, impair the maturation of autophagosomes, leading to an increase in cell mass and tumorigenesis, as reported in patients with breast, gastric, and colon cancers [58]. On the other hand, the higher mutation rates and genomic instability in the immortalized mouse kidney cell line resulted in aberrant chromosomal numbers due to autophagy inhibition [59]. In contrast to Beclin-1, there is evidence of other autophagic proteins that have an effect on tumor progression. Following this, mutation studies on ATG7 and ATG5 revealed an increased tendency for carcinogenesis, causing immune dysfunction and chronic inflammation. Knockout studies in mice have revealed the importance of other autophagic proteins as tumor suppressors [44]. Deletion of a segment of ATG7 and ATG5 from mouse liver cells experiencing oxidative stress caused liver carcinoma [60]. Consistently, other studies have indicated the importance of ATG3-ATG5 in cancer progression. Moreover, the deficiency of ATG4 proteins in mice increased their susceptibility to cancer infection [12]. Further, in an autophagy-deficient system in cervical cancer, accumulated ROS provided evidence of autophagy as a scavenging pathway that relieves the cell under stress conditions [61]. Also, it reduced inflammatory responses by acting as a negative regulator of the NLRP3 inflammasome in fetal colon cells [62]. Other studies have indicated the existence of anti-cancer medications that control the autophagic process. Hence, autophagy-regulated therapy can be employed to target cancer cell death. Altogether, these findings suggest autophagy is an indispensable pathway for tumor suppression, and impairment in the functioning of any autophagy genes may lead to cancer development.

### 3.2. The Role of Autophagy in Tumor Progression

Cancer cells display the ability to involve themselves in metastasis, which is the infiltration of local tissues via the vascular system. Metastasis is a process in cancer that helps tumor cells encounter increased mobility to migrate to different sites. The process is achieved in multiple steps involving the migration of cells from the primary tumor origin, where they enter the bloodstream and colonize the distant site upon exiting the vascular cells [63]. Various stresses in the primary tumor cells stimulate the autophagic pathway in order to protect them from inflammatory-associated death. As discussed earlier, the mechanism of autophagy is dependent on the stage and severity of cancer progression. In the early stages, it demonstrates a positive role that checks necroptosis and restricts their migration to distant sites. However, defects in autophagy lead to the accumulation of toxins and ROS generation, which results in DNA damage. This damage to the cells exerts a pro-tumorigenic role and promotes the survival of cancer cells in advanced stages of cancer [64].

Epithelial mesenchymal transition is the crucial biological phenomenon that causes tumor epithelial cells to attain a mesenchymal phenotype [65]. It includes loss of cell-cell adhesion leading to cancer metastasis, increased cell mobility, and the invasiveness of cancer cells [66,67]. Certain studies report the interconnection between the autophagic process and EMT in cancer. Upon nutrient deprivation, the process of autophagy supported the migration of HCC cells [68]. According to Catalano et al., it was noted that nutrient stress and mTOR suppression induced autophagy and reduced cell-cell migration in the cells of glioblastoma. Moreover, knockout experiments of autophagy-related proteins like ATG7, ATG5, and Beclin-1 enhanced the invasiveness of cancer cells in glioblastoma [69]. Autophagy also upregulates *HIF-1a*, which produces VEGF and is responsible for tumor vascularization in patients with pancreatic cancer [70]. The inhibition of the autophagic pathway by the shRNA cassette had been associated with impaired cell invasion but not with the proliferation of cells, as reported in the 3D model of glioblastoma [71]. Additionally, autophagy initiation supported the invasion of bladder cells by regulating the TGF-β pathway and EMT [72]. Furthermore, genetically-induced autophagy in Ras-activated cells inhibited the EMT process [73]. Interestingly, other studies established an inverse relationship between autophagy and cell migration in the cells of the primary tissue [69]. Intriguingly, the autophagy process can induce or inhibit the phenomenon of EMT and vice versa [74].

The mechanism of autophagy in cancer is also driven by the signaling pathways transduced by pro-inflammatory cytokine mediators. Of which, IL-6 and IL-17 are known to induce the expression of autophagic intermediates that lead to macroautophagy in lung cancer [75,76]. It induces transcription factors and upregulates inflammatory mediators essential to pyroptotic cell death [77,78]. On the other hand, it elevates autophagic proteins like LC3II and Beclin-1 to initiate the process of autophagy. These proteins achieve the elongation of the isolation membrane, resulting in autophagosomal maturation. The autophagosomes thus formed are key components in facilitating the autophagic process. All these events together drive the process of inflammation in tumor-associated macrophages, leading to an increased autophagy response in the cells of cancer patients [79]. In accordance with the role of autophagy in mediating inflammation and promoting cancer metastasis via canonical macroautophagic mechanisms, autophagy may serve as a therapeutic target in lung cancer.

## 4. Autophagy Describes the Tumor Microenvironment

As discussed in the above sections, there are multiple stressors that drive the autophagic mechanism. Moreover, the available literature suggests that autophagy modulates the tumor microenvironment via different signaling mechanisms [33]. Autophagy is an important physiological event that degrades macromolecules and abnormal protein components that meet the energy demands of nutrient-deprived cells to maintain cellular homoeostasis. It is also known to have a leading role in promoting angiogenesis. Through its ability to induce *HIF-1α*, it directs the formation of new blood vessels. It has been demonstrated that ATG5 regulates stress-induced angiogenesis through the HMGB1 pathway [80]. STAT3 is a major transcription factor that induces *HIF-1α* in cancer-associated fibroblasts and also elicits VEGF production to favor tumor development [81]. In contrast to cell survival under stress conditions, the mechanism of autophagy supplies the local tissues with plenty of nutrients [82]. In cancer, the fibroblasts mature into myofibroblasts, attaining the CAF phenotype. Recent studies shed light on the importance of signaling mechanisms in providing nearby tumor cells with the necessary signals to favor tumor development. Co-injection of cancer cells and cancer-associated fibroblasts expressing pro-autophagic molecules into immuno-compromised mice promoted tumor growth and metastasis in lung cancer cells [80]. This study reports the role of CAFs in providing a fertile microenvironment for tumor cells [83]. However, it is unclear about the role of autophagy in sustaining cancer cells under nutrient deprivation. Current research supports the role of inflammation in uncontrolled cell proliferation and oncogenesis. Autophagy is supposed to be a key player in inflammatory responses in lung cancer. However, its suppression is linked to increased necrosis. Previous studies have indicated impairment in the autophagic process and apoptotic mechanisms that induce necrosis, accelerating tumor growth via inflammatory reactions. All of these results suggest that autophagy is crucial for cell death and inflammation brought on by necrosis [84].

With time and in later stages of tumor development, tumor cells induce specific physiological changes within the host tissues. The combination of cells and cytokines produced through the signaling cascade constitutes TME [85]. The components of TME depend on the type of tumor, but distinguishing qualities include immune cells, vascularization, and extracellular matrices. According to some experts, TME is believed to be an active factor in the development of cancer that contributes to tumor survival, localized infiltration, and metastatic propagation. It is known to regulate low oxygen and nutrient stress through angiogenesis to combat a hypoxic environment [86]. NFkB, which is a master regulator, is stimulated through multiple signaling mechanisms to favor tumor growth, which further results in tumor escape. Tumor escape is a condition that inhibits the natural tendency of immune cells and promotes the apoptosis of cells inhibiting tumors. In order to suppress the phenomenon of tumor escape, it is crucial to dive into its cellular and molecular understanding. Also, designing novel strategies against suppressive microenvironments can favor pro-tumor activity [87].

Autophagy is a conserved cellular defense process that directly drives host inflammatory events. Cancer, being a great example of inflammation, reflects the dynamicity and unbalance of pro- and anti-inflammatory cytokines. At various stages of tumorigenesis, cancer-associated inflammation leads to genome modification, increased cell proliferation, anti-apoptotic mechanisms, and an angiogenic effect [88]. Previous studies believe immune cells are a major force in causing cancer inflammation. On the basis of their mechanism of action and their role in cancer development, immunocytes are categorized into two subsets: the innate and adaptive immune subsets. On one side, innate immune cells refer to the subsets that generate a host immune response and recognize cancer antigen irrespective of the type of cancer and specificity. On the other hand, adaptive immune responses are generated as a result of lymphocytes unique to the type of cancer [89]. In the next section, we will discuss a detailed account of each of the immune arms and their intrinsic ability to mitigate tumor growth and progression.

## 5. Immune Cells as Modulators of the Autophagy Mechanism

**Figure 3:**
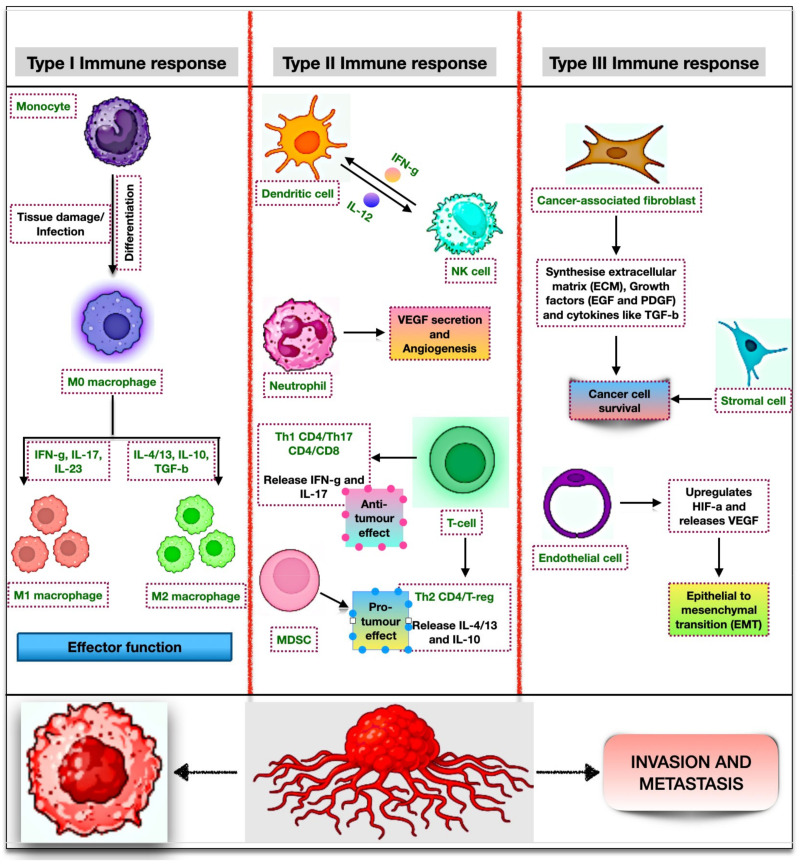
**Immune cells and their significance in lung cancer:** As discussed earlier, the immune response generated during inflammatory conditions is a result of different immune subsets. In general, immune responses are divided into three major groups. This figure precisely defines the functioning of immune cells and their primary role in cancer. Also, an increase in the number of anti-inflammatory subsets causes the tumor to progress and undergo metastasis.

In this review, we focus on the functional aspects of every immune cell, with a special focus on the signaling axes driving tumor progression. We also talk about how TME shapes every anti-tumor element to promote the growth of the cancer. Also, for an understanding of the primary role of immune cells during tumor development, refer to Figure 3.

### 5.1. Immune Cells

Broadly, the immune response in cancer is classified on the basis of the immune regulators driving the inflammatory condition and is categorized into Type I, Type II, and Type III immune cells. Type I immune cells include monocytes and macrophages that play a key role in tissue repair and cellular homeostasis. However, dendritic cells, natural killer cells, myeloid-derived suppressor cells, T-regs, and T-cells display great significance in generating Type II immune responses in cancer. Type III immune cells include subsets specific to the state of lung cancer, like stromal cells, endothelial cells, and cancer-associated fibroblasts.

#### 5.1.1. Macrophages

Macrophages are the cells of the innate immune system that polarize from circulating monocytes upon tissue damage. After differentiation, macrophages are able to detect and respond to infection, exhibiting a critical role in tissue homeostasis and healing [90]. Being an essential driver of cancer-associated inflammation, their relevance has been described at every step of cancer development [91,92]. On the basis of the evident roles of macrophages in inflammation, they are of two types: Type I macrophages (M1 macrophages) and Type II macrophages (M2 macrophages). The former, being pro-inflammatory in nature, generates immune responses with the aim of promoting wound healing via the synthesis of pro-inflammatory mediators like IL-6, TNF-α, IL-1β, and other cytokines [93]. Unlike M1 macrophages, M2 macrophages are also referred to as tumor-associated macrophages. M2 macrophages, being anti-inflammatory in nature [94,95], represent the cytokine pool of other anti-inflammatory subsets like MDSCs [96] and T-regs [97]. All these cells exert an immunosuppressive function and play a major role in cancer-mediated inflammation through the production of TGF-β and IL-10 in host cells [98,99,100]. According to other studies, TAM exerts an immunosuppressive role in tumor progression and angiogenesis through the production of EGF [101] and VEGF [102], respectively. It is also supposed to remodel the extracellular matrix by releasing MMPs upon activation [103]. A variety of chemokines, including MCSF [104] and VEGF [105], recruit TAMs to the site of cancer inflammation. Also, every signaling cascade happening in TAM activates the molecular mechanisms of macroautophagy, resulting in chronic inflammation [106].

The polarization of monocytes into the M1 phenotype employs the activation of the CCR2 receptor [107] to form IL-1β [108] and IL-23 [109]. This transition is also facilitated by the IL-17 [110] and IL-23 [111] signaling processes with the aim of activating NFkB, which is required for the translation of a pleiotropic IL-6 cytokine. In contrast, the effector function of M2 macrophages is brought upon by anti-inflammatory mediators like IL-4, IL-13 [112], and MCSF [113] generated by immune cells. Besides this, other signaling pathways, such as IL-6 [114] and IL-17 [115] are activated to ensure the formation of proteins involved in TAM’s survival and proliferation. The transcription factors activated as a result of this signaling cascade are involved in triggering the autophagic events that cause macroautophagy [116,117].

#### 5.1.2. Dendritic Cell

DCs are professional APCs and, hence, play a crucial role in the presentation of an antigen to mount an immune response. Due to its diverse role in the immune system, DC links both innate and adaptive immunity [118]. The cytokine milieu of TME decides the fate of DC by supplying environmental factors to generate either a pro-tumor or an anti-tumor effect. Mostly, during the later stages of tumor development, it has been viewed as tolerating the effects of immunosuppressive cytokines. DCs have been shown to have an indirect effect on autophagy initiation via the production of IL-12; it is a heterodimeric pro-inflammatory cytokine that stabilizes the NK cell [119] and Th1 phenotype [120] to secrete IFN-γ [121]. This initiates a series of sequential events that induce the expression of autophagic proteins and the activation of the canonical mechanism of autophagy [122].

#### 5.1.3. Natural Killer Cell

The cytotoxic ability of NK cells to recognize transformed and virally infected cells makes them a potent immune subset [123]. The role of NK cells in cancer progression relies on the activation of their IL-12 receptor, which stimulates the production of the pro-inflammatory cytokine IFN-γ via the stimulation of STAT 4. Lymphokine thus released has a varied role in the differentiation and stabilization of the Th1 phenotype. Also, previous research has confirmed the anti-tumor effect of NK cells, which has been observed by the effective killing of tumor cells that are in the bloodstream, preventing metastasis. It is evident that these cells produce the cytotoxic components, perforin and granzyme, to destroy tumor cells and initiate apoptotic mechanisms [124] through the release of inflammatory mediators like TNF-α [125].

#### 5.1.4. Neutrophils

Neutrophils are recognized as the first immune cells to recruit at the site of inflammation. They are understood to play a primary role in pathogen clearance via phagocytosis of bacterial components. Depending on the type of tumor and its role in cancer, TANs are classified into N1 and N2 subtypes to represent anti-tumor and pro-tumor states, respectively [126]. The recruitment of polymorphonuclear cells into TME is assisted by stromal cells secreting CXCR2 ligands [127]. This promotes neutrophils to facilitate nascent inflammation in tumor sites. Moreover, it has been shown to enhance invasiveness, vascularization, and the proliferation of cancer cells with the secretion of VEGF and MMPs, as reported in mouse models of lung and pancreatic cancer [128].

#### 5.1.5. Myeloid-Derived Suppressor Cells

MDSCs refer to an anti-inflammatory subset that belongs to a myeloid lineage supporting chronic inflammation. The recruitment of myeloid cells to the cancer site depends on the secretion of growth factors by tumor cells. Although the differentiation of MDSCs into M-MDSC (monocytic lineage) and PMN-MDSC (granulocytic lineage) varies with the tumor type, their recruitment to the inflamed site is basically mediated by the same variables that control the migration of granulocytes like neutrophils and monocytes.

Cytokines belonging to the STAT family, like STAT 1, STAT 3, STAT 6, and Erk1/2, maintain the MDSC phenotype. Its activation is dependent on the synthesis of IL-1β, IL-4, IL-13, and VEGF by different carcinoma and immune cells. Among all the transcription factors, STAT 1 and 6 are known to expand MDSC by regulating the production of suppressive proteins. All these proteins produced as a result of the signaling cascade stabilize the immunosuppressive phenotype of MDSC, together with other immunomodulatory cells such as TAMs and T-regs [129]. Subsequently, upon activation, M2 macrophages initiate other pathways to elevate macroautophagy, which leads to the expression of autophagic intermediates [130]. Altogether, the data reveal the importance of MDSCs in achieving M2 homeostasis, which triggers the phenomenon of macroautophagy. Therefore, targeting specific transcription factors and signaling intermediates may contribute to overcoming the autophagic pathway and, thereby, suppressing tumor development.

#### 5.1.6. T-Cells

T-cells are the principal players that orchestrate the adaptive immune system. Depending on the immunological background, T-cells can achieve either a pro-tumor or an anti-tumor phenotype. These cells are actively researched in various cancer types since they are the second most common immune cell type to be discovered in human tumors after TAMs. The primary role of T-cells in early tumor development is well established by their migration in the TME and their differentiation into effector T-cells to get rid of tumor antigens [131]. Histopathology analysis of human tumors has confirmed the extension of tumor-associated T-cells in the hypoxic environment [95]. In many malignancies, the differentiation of tumor-infiltrating Th1-related cytokines and cytotoxic (CD8+) T-cells is associated with a favorable outcome for long-term survival and cancer-free progression [132].

If T-cells are highly constructive in mounting an antigenic response, how come cancer cells conquer the effect of thymic cells? According to preclinical examinations in cancer patients and mouse models, it has been reported that tumor cells use the immunoregulatory characteristics of T-cells while impeding their anti-cancer activity, like tumor infiltration and cytotoxic response [133]. There are various different T-cell populations inside the TME that have an impact on carcinogenesis. CD8+ T-cells potentiate the killing of abnormal tumor-associated antigens. Apart from cancer’s cytolytic response, CD8+ T-cells suppress angiogenic activity through IFN-γ secretion [134,135]. Within the TME framework, CD4+ T-cells develop into distinct subtypes and integrate an extensive set of immunological responses. T helper 1 (Th-1) cells are CD4+ T-cells that promote inflammation and aid CD8+ cells [136] by secreting IL-2 and IFN-γ [137]. In several forms of cancer, higher Th1 cell counts within the TME are linked to favorable outcomes. The differentiation of CD4+ T-cells into a Th17 phenotype depends on IL-23 and TGF-β signaling that upregulates the transcription factors RORγt and STAT 3 for the production of IL-17 cytokines [138]. The immunomodulatory state of T-regs is acquired to reduce the inflammatory response of pro-inflammatory cytokines. These suppressive cells release immunoregulatory cytokines and dampen anti-tumor activity to promote M2 homeostasis, which lessens the pro-tumor effect of T-cells forming protein molecules that influence inflammatory responses and elevate autophagy [139].

#### 5.1.7. Type III Immune Cells

As mentioned above, stromal cells, endothelial cells, and cancer-associated fibroblasts constitute the type III immune responses in cancer. In order to facilitate crucial phases in tumor growth, cancer cells recruit founder cells from the local endogenous stromal tissue. The composition of stromal cells varies between different tumors and consists of CAFs, vascular endothelial cells, stellate cells, and adipocytes. When stromal cells are attracted to the TME, they release a variety of substances that have an impact on the malignancy of transformed cells.

In the TME, endothelial cells assist in coordinating the development of new blood vessels. In addition to its primary role, the vascular endothelium also transports immune cells, maintains metabolic equilibrium, and supplies water and nutrients to the growing tumor mass. Cancer cells utilize passive diffusion for exchanges of gases and the transfer of nutrients during the early stages of tumor growth. While tumors are 1–2 mm^3^ in size, the TME becomes hypoxic and acidic due to a lack of oxygen and an accumulation of metabolic waste. To overcome an insufficient oxygen supply, cancer cells form new blood vessels. This leads to the activation of *HIF-1α* in response to low levels of oxygen. In particular, *HIF-1α* causes endothelial cells to produce pro-angiogenic factors like EGF, PDGF, and VEGF.

EC have a great degree of plasticity, can alter cell fate, and apparently undergo endothelial-to-mesenchymal transition to achieve the CAF phenotype. Proteins like TGF-β and BMP regulate the transformation process and the invasive nature of fibroblasts in lung cancer. CAFs are regarded as a key player in furthering the crosstalk between cancer cells and the TME. For example, the existence of CAFs is significantly associated with breast and lung cancer patients. It is also shown to synthesize a majority of growth factors, extracellular matrices, and cytokines to shape the TME. In order to further the migration of cancer cells, they secrete MMPs and TGF-β to promote the angiogenic process. On the whole, CAFs release immune-modulatory chemokines to facilitate an immunosuppressive condition [86].

## 6. Inflammasome as a Crucial Regulator in Autophagy and Cancer

Cancer development is a complicated process that implies tumor cell internal and external signals, both of which are essential for cell malignancy [140]. For a long time, the association between inflammation and the development of cancer has been known, and the inflammatory response is understood to be crucial to the onset and development of cancer. The inflammasome, which is a host’s innate multiprotein complex, was initially used to describe mechanisms facilitating pro-inflammatory events against pathogens. NLRP3 is the most widely studied inflammasome and has been linked to the etiology of several disorders, including cancer. Being an important player in the pathogenesis of cancer, the NLRP3 inflammasome comprises three crucial components: the NLRP3, the ASC (which consists of the CARD domain), and pro-caspase 1 (which bears effector functions). Three domains constitute NLRP3: an N-terminal pyrin domain (PYD), which interacts with the ASC’s pyrin domain to assemble the inflammasome; a NACHT domain responsible for inflammasome activation; and a C-terminal leucine-rich repeat (LRR) domain that does not have much significance in inflammasome assembly. ASC activates autoproteolytic caspase-1 by binding pro-caspase 1 through their common domains. Caspase-1 releases inflammatory cytokines like IL-1β and IL-18, which also cleave the protein GSDMD, causing pyroptosis, a type of programmed cell death [141].

Tumoral subsets, including MDSC and TAMs, are known to release IL-1β and induce CAFs to mediate inflammation [142]. In addition, a recent study reported its role in promoting an immunosuppressive environment in the lung [143]. The production of IL-1β by cancerous cells and TAMs has been shown to enhance the tumorigenic activity of CAFs, indicating IL-1β is an effective driver to expedite tumor development [144]. Furthermore, IL-1β has been shown to have autocrine signaling that directly favors cancer progression [139]. Research by Daley et al. has provided insights on how NLRP3 proceeds to host inflammation and produce anti-inflammatory cytokines upon the differentiation and activation of an immunomodulatory subset. In the same study, the predominant role of NLRP3 was associated with the differentiation of T-cell subsets into Th2, Treg, and Th17 cells, facilitating IL-10 production [145]. Also, the pivotal role of the IL-6-STAT3 inflammatory loop has been linked with the genesis of MDSCs and their ability to induce immunosuppression [146]. This suggests a significant role for NLRP3 in lowering the anti-tumor response that promotes immunosuppression in patients with lung cancer [147,148]. A recent study in a mouse model of NSCLC showed that the cells of the myeloid lineage produced IL-1β irrespective of inflammasome activation, which suggests the infiltration of other immune cells to tumor sites [149]. In adenocarcinoma lung cancer patients, the extended role of IL-1β was shown to enhance expression levels of angiogenic molecules [150]. Also, LPS, which is a tumor antigen, induces the formation of lung tumors upon NLRP3 activation [151]. In line with this study, it is clear that the transcription factor NFkB drives the recruitment and activation of the assembly that causes inflammasome formation [152]. Therefore, it is essential to neutralize the effect of NFkB so as to overcome the pro-tumor activity of the NLRP3 inflammasome [153].

Autophagy is a physiological defense mechanism regulated by multiple proteins and plays a pivotal role in nutrient recycling [154]. It is believed to play an essential role in several life processes, from cell development and differentiation to ageing. Besides the role of autophagy in maintaining homeostasis, studies indicate a relationship between the autophagy pathway and inflammatory events that form the inflammasome [155]. In 2008, Saitoh and his group were the first to demonstrate the relationship between autophagy and inflammation, where autophagy negatively regulates inflammasome formation [156]. However, other studies have discussed the role of autophagy in inducing NLRP3 activation. It has been discovered that NLR domains interact with autophagy proteins, providing a method for direct NLR regulation of autophagy [157]. According to previous studies, certain NLRs, both those that form inflammasomes and those that do not, may engage in NACHT domain-mediated interactions with the protein Beclin-1, which is essential for the start of autophagy [158]. Additionally, other studies have suggested a role of NLRs in promoting the formation of autophagosomes [97].

**Figure 4:**
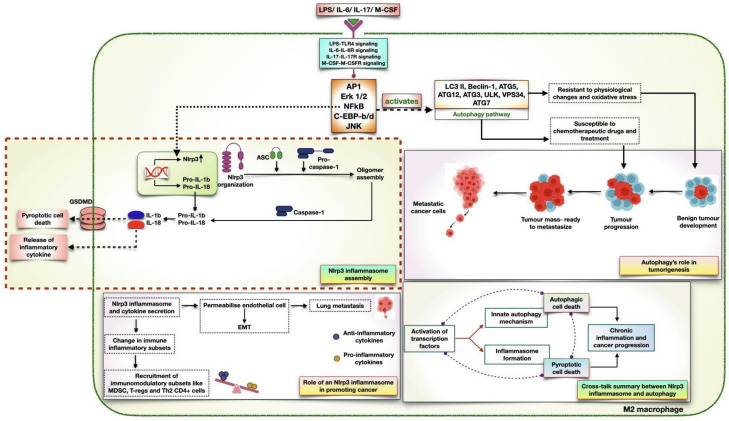
**Inflammasomes as modulators of autophagy mechanisms:** Inflammasomes are multi-protein complexes that drive host inflammation. The process of inflammasome formation starts with various different ligands. Upon specific interaction between ligand and receptor, several transcription factors get activated that may have a direct or indirect role in cancer progression. Among all the transcription factors, NFkB assembles the inflammasome. Simultaneously, autophagy is activated to relieve cellular stress. As a result, chronic inflammation occurs, which results in macroautophagy.

Interestingly, the role of NLRP3 in inducing autophagy was observed upon macrophage infection with *Pseudomonas aeruginosa*. The finding was consistent with the significant expression of LC3II, which suggests the inflammasome’s role in driving autophagic events [157]. Despite this, the role of signaling pathways in transducing inflammatory events remains unclear. Among the list of cytokines, IL-6 [159], IL-17 [160], M-CSF [161], and tumor antigen, LPS [162] are essential for stimulating transcriptional activators responsible for inflammasome assembly and autophagy activation. Although these cytokines employ different signaling cascades, the activation of numerous transcription factors, including JNK [163], NFkB [164], Erk1/2 [165], AP1 [166], and C/EBP-b/d [167], showed promising results in NLRP3 inflammasome formation and autophagy initiation (Figure 4). However, certain questions remain unresolved regarding the crosstalk between innate immunological processes to determine the potential target for autophagic proteins favoring cancer development. Therefore, to decipher anti-NLRP3-based cancer drugs, it is important to unravel the molecular mechanisms that promote immunocompromised conditions and initiate autophagy, promoting cancer cell survival.

### Decisive Role of IL6-IL17-IL23 Axis in Autophagic Cell Death

IL-6 is a multi-functional cytokine that has a pleiotropic role in different immune activities such as cell proliferation and differentiation, regulating its physiological role. It exhibits both pro-inflammatory and anti-inflammatory functions through the type I receptor complex associated with the signaling component, gp130. Under controlled conditions, it activates various signaling events crucial for cell survival and development. However, its deregulation is associated with the onset of classical and trans-signaling events facilitating chronic inflammation and with the pathophysiology of many inflammatory diseases like cancer, rheumatoid arthritis, multiple myeloma, AIDS, and many more [168,169]. Through its role in mediating immune regulation, it is an important target for disease control. In contrast to IL-6, IL-17 was discovered as a member of the IL-17 family known to mediate the secretion of pro-inflammatory cytokines via immune subsets. This family includes six structurally related cytokines, IL-17 A-F (A, B, C, D, E, and F), of which IL-17 A is involved in the progression of the inflammatory state of cancer and other autoimmune diseases. IL-17 is a pan-cytokine marker of CD4+ Th17 cells, which have markedly increased expression of a transcription factor, RORγt, which activates STAT3 to control and stabilize IL-17A production. It has been reported that T-cells have a prime role in maintaining Th17 attributes through their ability to produce IL-23 cytokines and sustain the Th17 cell lineage. IL-23 belongs to the IL-12 family of cytokines, and its evolution is linked to progressive inflammation. In addition, the combination of the anti-inflammatory cytokine TGF-β and IL-6 has been observed to support IL-17 secretion in a similar way as IL-23 does. With the reported evidence, we can therefore summarize the major contribution of IL-23 together with IL-6 to nurture the phenotype of the IL-17 family, specifically IL-17A [170,171]. During an inflamed state, CD4+ cells behave as major cytokine producers, releasing multiple cytokines. To a lesser extent, other accessory immunocytes control and regulate cytokine production. In line with this, IL-6 is produced by monocytes, tumor cells, CAFs, endothelial cells, and cells specific to a diseased state [172]. However, T-cell variants, including CD4, CD8, and other immune subsets, generate IL-17A. The production of IL-23 has been reported to occur through DCs and M1 macrophages [111,173]. Since the obvious roles of these cytokines in progressing a diseased state can be targeted to rescue tumor development, it helps to regress progressive inflammation.

The significance of these cytokines relies on the transduction of a signaling cascade that activates different cellular mechanisms. Among these, IL-6 executes classical and trans-signaling mechanisms to generate inflammatory responses in accordance with the cellular environment. Both of the signaling events initiate three pathways: the JAK-STAT pathway, the RAS-MAPK pathway, and PI3K signaling [174]. Focusing on the insights into lung cancer, the production of IL-6 in lung TME actuates a signaling response in T-cells, M2 macrophages (TAM), and Type III immune cells, including CAFs and endothelial cells. In T-cells and Type III cells, the binding of IL-6 triggers JAK1/2-STAT3 activation, which plays a key role in activating two different key events. In T-cells, as discussed above, STAT3 expresses RORγt, which differentiates CD4+ T-cells while maintaining the Th17 lineage. The proliferation of Th17 cells is associated with the expression of IL-17 and IL-6, suggesting the autocrine role of IL-6 in mediating inflammation. Besides driving inflammatory events, the role of STAT3 has been proposed to upregulate the expression of *HIF-1α*, causing chronic inflammation through VEGF production. This is in line with the chief role of IL-6 in driving disease states during cancer development. To further understand the role of IL-6 in mediating cell death events, it is of greater importance to know at which state and through which component it triggers autophagic intermediate expression. As a result of PI3K/Akt signaling, IL-6 in M2 macrophages activates the canonical pathway of autophagy, leading to disease progression. This is assisted by NFkB expression, which is known to have a dual role in cancer. Primarily, it regulates the Nlrp3 gene, producing the protein form required to assemble the inflammasome. On the other hand, NFkB activates several autophagic intermediates like LC3II, Beclin-1, ATG5, ATG3, ULK, and ATG12. Since its role in mediating autophagy is associated with cancer cell survival, this also reflects IL-6’s role in linking inflammation and autophagy. Similarly, the activation of the MAPK pathway via IL-6 facilitates Erk activation, which upregulates the expression of proteins related to autophagy. Altogether, the above facts suggest the intricate role of IL-6 in assisting innate immunological responses in cancer development.

As aforementioned about the production of IL-17 by T-cells, it has a pronounced signaling effect in monocytes, M2 macrophages, and Type III immune cells. In monocytes and Type III cells, the binding of IL-17 through its receptor activates the TRAF6 pathway, which leads to the ultimate activation of NFkB. Once expressed, it translates IL6, which will have further significant roles in autophagy activation (refer above). In Type III cells, apart from sustaining cancer cells in a nutrient-deprived environment, the role of NFkB is vital to the proliferation, survival, and migration of cancer cells. It is achieved through cyclin D1 protein expression (cell cycle protein), IL4/IL13 secretion (maintains a reducing environment), VEGF production, and the expression of anti-apoptotic proteins like Bcl-2 and Bcl-xL. In TAMs, the role of IL-17 is expanding. Similarly, it activates the pathways, as it did in the above two cells. Besides this, it activates various transcription factors that have a pronounced effect on autophagy activation. Through IL-17, TFs like Erk, CEBP-b/d, AP1, and NFkB, along with the MAPK intermediate MKK4/7, get activated. NFkB activates the same autophagy proteins as we discussed for IL-6 signaling. Erk and CEBP-b/d take charge of LC3II and Beclin-1 activation. AP1 is responsible for LC3II, ATG7, ATG5, and Beclin-1 activation. MKK4/7 is an indispensable intermediate that activates autophagy through two different mechanisms. Firstly, it activates JNK, which is important to dissociate the Beclin1-Bcl2 complex and trigger autophagic events. As JNK separates Beclin-1, it relieves the inhibitory effect of Bcl-2, which is responsible for dictating macroautophagy in cancer-infected cells. Next, upon JNK activation, it interferes with cell death mechanisms through VPS34, ULK, LC3II, and ATG7 expression. After having discussed the diverse role of IL-17 in transducing signaling mechanisms, we can conclude that IL-17 plays a pivotal role in harnessing autophagic events to promote cancer cell survival.

Lastly, the role of IL-23 in promoting autophagy is important. It is seen to be produced through M1 macrophages and has an effector role in monocytes and T-cells. In monocytes, IL-23 activates STAT3 through JAK2/TYK2 activation. The result is the production of IL-6, which translocates to M2 macrophages to drive cell death mechanisms. In T-cells, it acts as a mediator in differentiating Th17 cells and generating IL-17. Again, as discussed above, the generated IL-17 plays a multi-functional role in driving autophagic cell death and achieving inflammatory responses in cancer. Therefore, to sum up the above statements, we can conclude the importance of the IL6-IL17-IL23 signaling axis in initiating innate autophagic mechanisms. And it also suggests the role of these cytokines in mediating inflammasome assembly in a cancerous state. Hence, to achieve a novel therapeutic strategy, targeting the immune signaling axis would help overcome cancer development and improve disease outcome.

## 7. Autophagy and Drug Resistance in Lung Cancer

It is interesting to note that various chemotherapy medications may have opposing effects on autophagy, leading to either cell death or cell survival [175]. In cancer cells, autophagy is triggered under stress as a result of gene mutations, epigenetic alterations, or an imbalance in the cell’s ability to regulate its own growth. Over the last ten years, the role of autophagy as a protective mechanism has been strengthened to facilitate cell survival during chemotherapeutic treatments. The increased resistance towards anti-cancer medications is seen to be offered by the multi-drug resistance protein 1 (MDR1), which belongs to a class of ATP-binding cassette transporters [176]. As per the literature, the concentration of autophagic proteins was found to be higher during chemotherapy, suggesting a link between autophagy and MDR.

It is obvious that several studies have shown a strong association between autophagy and drug resistance in NSCLC. Moreover, other studies have highlighted the significance of multiple signaling pathways, including the MAPK pathway [177], PI3K/Akt/mTOR [178], Wnt signaling [179], and p53 signaling [180], in causing cancer cell resistance. Additionally, these signaling events have been shown to upregulate the expression of autophagic intermediates, resulting in autophagy. The development of resistance to EGFR inhibitors has long been a significant clinical problem. In NSCLC cells mutated with EGFR, erlotinib induces both autophagy and apoptosis, but blocking the autophagy process can increase erlotinib’s cytotoxicity to cancer cells. In vitro studies revealed the activation of autophagy mechanisms and LC3 expression upon EGFR resistance in NSCLC patients [181]. According to a study by Wu T. et al., autophagic events elevate cisplatin resistance in lung cancer cells. In contrast, the inhibition of autophagic mechanisms weakened cisplatin-generated drug resistance [182]. Based on the above findings, researchers postulate autophagy as a defense mechanism against cancer cells that contributes to the development of drug resistance in lung cancer patients. Understanding how autophagy contributes to drug resistance will make it easier to investigate ways to control autophagy to enhance cancer therapy. Due to the strong relationship between autophagy and drug resistance, autophagy will likely develop into an effective target in cancer treatment [183]. Also, combining autophagic therapy with the existing anti-cancer drugs will facilitate clinical trial outcomes.

## 8. Systems Biology and Its Potential Role in Targeting Autophagy

Systems biology is a tool that allows us to retrieve the convoluted network of biological systems by understanding the behavior of every molecule involved in a reaction. It entails a variety of methodologies to understand biological processes at the system level, including network reconstruction, analysis, and mathematical modeling [184]. The ability of a system to maintain its function in spite of extrinsic and intrinsic perturbations is defined as robustness [185]. Adaptation, on the other hand, refers to a system’s capacity to change its behavior in response to environmental changes [186]. Tumors are intricate structures that exhibit robustness and adaptation; therefore, they eventually develop resistance to anti-cancer medications [187]. Approaches to overcome resistance are one of the major goals and promises of systems biology. Through systems perspectives, researchers deepen their understanding at a molecular level and dissect signal transduction pathways to analyze the phenomenon of drug resistance and determine which medicine combinations works best for a particular tumor [188]. Therefore, it is important to model the functioning of the signaling network to establish cell-specific responses in molecular therapies.

Systems biology research seeks to comprehend the characteristics of a particular system, which in the context of cancer may include primary cells from the patient or tumor cell lines. Recently, systems biology findings from both SCLC and NSCLC have been used to identify diagnostic features and investigate novel therapeutic strategies for lung cancer [189]. Due to the significant contribution of NSCLC in developing cancer, a major research focus reveals the importance of the immune axis in driving autophagic mechanisms [11]. It is evident that several transcription factors, including NFkB, enhance the cell death events that favor tumor cell survival, whereas its inhibition might help to improve the clinical outcome [190]. With a better understanding of the autophagy system, it is possible to develop inhibitors against early or late autophagic events and stop specific cargo from entering autophagosomes in infected cells. At present, the chemotherapeutic drug treatments available against cancer have become resistant for patients [191]. With regard to the process of drug resistance in cancer, systems biology employs comprehensive research that can provide solutions to challenging issues about progressive cancer [1]. In addition, network biology, being a strong and valuable tool of systems biology, can be utilized to analyze biological networks and achieve precision medicine [188]. Therefore, it is of utmost importance to focus on intricate network connections between cellular components that can open a path for the discovery of resistance-associated drug targets and realize the potential for personalized molecular medicine.

After decades of conducting research focused on developing our understanding of the pathophysiology of clinical oncology, it is well accepted that any or all outcomes or symptoms in lung cancer are a combined effect of the molecular interactions in a cell. These cells organize to form a whole organism, thereby increasing the complexity of molecular entities from DNA to proteins. The complex diversity within a single cell expands researchers’ ability to define systems that can include factors in the context of location and time. It is unreliable that each biological component experiences the same environmental cues throughout. Instead, internal genome complexity is what provides flexibility and achieves a long-term survivability effect in multicellular organisms [192]. As per evolutionary history and individual evolution, the increased adaptation from a unicellular to a multicellular state resulted in an increased host’s adaptation in terms of microchanges in the environment. Understanding how to replicate this organized complexity in order to create predictive algorithms for future applications in healthcare is an integral part of systems biology [193]. For instance, a regulatory transcription factor, NFkB, may be a possible crosstalk between two complex phenomena such as autophagy and the inflammasome that drives cancer progression [190]. Therefore, targeting the common node may help reduce the system’s complexity in practical interventions and flourish in the era of personalized medicine for disease treatment and its control monitoring.

### Network Analysis Using Systems Biology

A network analysis expresses several elements, such as genes or proteins, and their interconnections. A gene or protein molecular network can be schematically constructed using nodes and edges. Genes, proteins, and drugs can all function as nodes in molecular interactions. However, edges are functional entities that connect to the gene regulatory network, protein interactions, and the mechanisms of drug inhibition and activation. The way in which the nodes and edges are arranged in a network can be explored to better understand biological systems [194]. In network biology, usually a few nodes are designated as hubs due to their large interconnections between them. The higher associations between nodes refer to modules that coincide with the biological entities of molecular components [195]. To further analyze the biological network, there are many significant tools that have been developed. One such method is network motifs, with which we can decipher the important signaling network underlying tumor development [196]. Additionally, according to Przulj et al., the use of protein-protein interaction networks may make it easier to unravel the transcription factors and/or important proteins that drive chronic inflammation in a diseased state [197]. Also, its application may turn out to be a good strategy for overcoming the phenomenon of cancer drug resistance.

Protein-protein interaction elucidates the interaction between proteins. To describe the nodes and edges in a network, proteins are termed nodes, whereas the interactions between them are referred to as edges [198]. For this, the data can be collected using different methodologies, including computational approaches, or from various resources such as STRING [199], HAPPI-2 [200], and Bioplex [201]. At present, PPI and its functioning are widely accepted as facilitating high-throughput modeling of inter- and intra-cellular signaling. This approach has been frequently adopted in other research to determine differential gene expression from the transcriptome of diseased patients while extracting a gene set from the other databases. Today, the use of networks has advanced to model intercellular associations with scRNAseq data. To understand how autophagy assists cancer, scRNAseq data can be obtained from healthy subjects and lung cancer patients to develop a PPI-based network [202]. This can be achieved by identifying a proper network through visualization of ligand-receptor interactions and the transduced signaling events with respect to a particular ligand [203,204]. The differential gene expression can be compared between two different groups, and genes that are highly expressed among them can be considered cell markers. Using the selected candidate, the building of a cell-cell interaction network can be executed, which can be further validated through statistical significance. The network thus built helps in establishing the direct and indirect relationship between immune cells and lets researchers know which immune subset plays a key role in advancing cancerous states through modulating the cellular cytokine environment [205]. Besides that, it informs us of the differences in autophagic protein expression between cancer patients and the NHS. With this evidence, it will be simpler to churn out the common node and the unregulated proteins during the different stages of autophagy.

Another domain under network biology is the construction of metabolic networks, wherein metabolites represent nodes and enzymes are edges [198]. By determining the metabolites’ flow across the network in steady-state, flux-balance analysis is the method most frequently used to analyze metabolic networks [206]. The goal of the study is to maximize the outcome of a particular reaction by identifying the optimal potential flux through the numerous reactions. Cell mass or ATP production are typically used to illustrate these reactions. Recently, it has been possible to access the metabolic networks of complete species. In order to simulate the human host, the Recon2 programme offers a thorough global reconstruction of human metabolism [207]. In lung cancer, the significance of constructing a metabolic network implies the fact that it ensures evaluating the metabolic profile of cancer cells and at what level it differs from untransformed cells [208]. The network has been known to have a higher degree of interaction completeness, which makes it a perfect tool for modeling [209]. However, because RNA sequencing is now much more high-throughput, metabolomic investigations are much less common than transcriptomics research. To determine whether metabolic networks could serve as a potential biomarker for predicting treatment response in NSCLC, more research is required.

Yet another important arm of network biology is the Gene Regulatory Network, which includes molecules guiding gene expression. In GRNs, nodes refer to any transcription factor, cis or trans-regulatory element, or miRNA, whereas edges refer to physical interactions between molecules [198]. To map GRNs, techniques like ChIP, DNA affinity purification, and ChIP sequencing can be adopted [210]. The modeling of GRNs can be completed using Bayesian network approaches that employ the Bayes theorem to understand the interdependent expression of two different genes [205]. The expanded use of GRNs in lung cancer may be used to figure out the influence of autophagic proteins on cancer-associated transcription factors. With this, it will be easier to identify the gene sets responsible for a diseased state, and targeting such genes would be equally important to establish anti-cancer treatment. Unlike GRNs, gene co-expression networks (GCNs) can be exploited to explore cytokine signaling [211] in lung cancer patients. Genes here represent nodes; however, co-expressed genes are edges [198]. According to a study with a focus other than cancer, the transcriptomic profile of biopsies from healthy and unhealthy subjects investigated the role of IL-23 in the pathogenesis of a diseased state due to its pro-inflammatory nature [212]. Similarly, to determine important molecules and the role of key players like IL-6 [114], IL-17 [213], and IL-23 [214] in progressing NSCLC, the cytokine profiles of various immune cells (as discussed above) can be studied. By analyzing the cytokine profile, the ratio of pro-inflammatory to anti-inflammatory subsets can be evaluated to determine which cells are more likely to have a shift towards tumorigenesis [215]. This also supports the hypothesis that inflammatory diseases, including cancer, TB, HIV, IBD, and others, reflect a pathogenic myeloid-cell lineage that creates immune-suppressive conditions, as unraveled through various studies [216,217,218].

To understand the cell-signaling network, multi-layered networks are much more focused under network biology. Over the last ten years, it has become popular to collect multiple omics data types from an individual sample because it is believed that doing so could give researchers deeper insights into the biology of disease. The emergence of multi-omics requires the fusion of several network modeling strategies, which have been required to unravel the crosstalk between autophagy and cancer [219]. A cell signaling network consists of two components: an upstream component, which is a PPI network and contains several intracellular events, and a downstream element that includes gene regulatory networks and transcription factor-targeted interactions [220]. With the application of specific algorithms to the cell signaling network of infected individuals, it is possible to detect the signaling cascade confined to a particular infection [221]. Therefore, by adopting a novel network biology methodology, researchers will be able to discover disease-associated genes in subgroups of LC.

**Figure 5:**
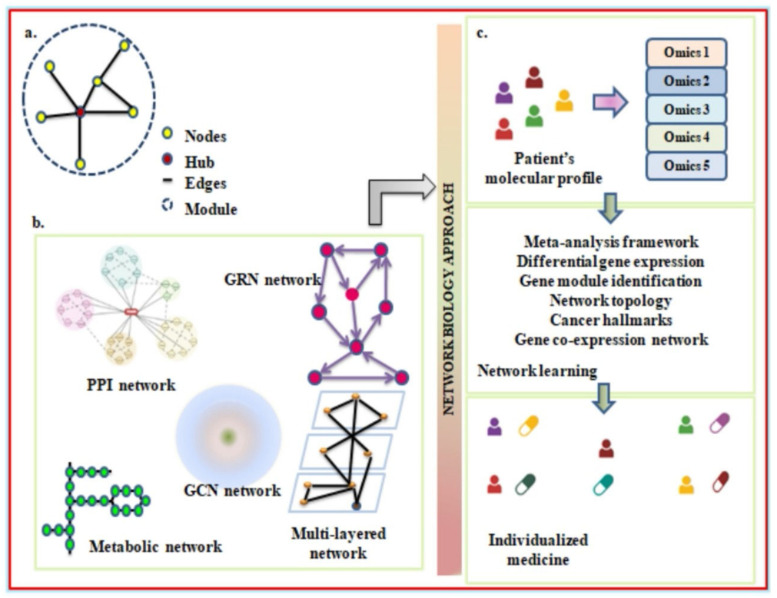
**Network biology and its approaches in targeting cancer** (**a**) Defines the overview of a network that includes nodes, hubs, edges, and modules. (**b**) Overview of different network biology tools to penetrate deeper into lung cancer research. (**c**) Includes methodologies through which patient data can be individualized for precision medicine.

Despite recent advances in elucidating cancer pathogenesis using the aforementioned techniques of network biology, there are still a number of obstacles to be overcome before precision therapy in lung cancer may be fully realized [222]. Previously, network biology models could only reconstruct biological networks using data like PPI networks, differential gene expression, or transcriptome data [223]. But recent advancements in cancer research show that multi-layered networks are more likely to produce potent and applicable insights for complicated diseases [192]. A study conducted on colorectal cancer reveals that the type, number, and location of immune cells may serve as a more accurate prognostic tool [224]. To date, a key challenge that will need to be explored in the aforementioned approaches is how to well integrate the enormous volumes of multi-omics data produced from various sources (Figure 5) and consequently yield therapeutically significant insights into lung cancer. Therefore, achieving the objective of precision medicine in cancer would require integrating patient genomes, transcriptomics, metabolomics, epigenomics, and metagenomic records together with histopathology and clinical information throughout time to maximize the potential of network biology [205].

## 9. Autophagy as a Therapeutic in Cancer Development

In this review, we have detailed the role of autophagy as both a tumor promoter and an inhibitor. In order to improve cancer immunotherapy, it is crucial to control autophagy. Autophagy is a physiological cell death mechanism that supplies nutrients to the inner cell mass of tumors. Being a double-edged sword, it develops cell resistance to chemotherapeutic drugs. Various studies, including one by Boya et al., suggest the stimulation of apoptosis upon autophagy inhibition. This implies the fact that lowering the expression of autophagic proteins promotes the expression of pro-apoptotic molecules, resulting in the programmed death of cancer cells [225]. Therefore, it is essential to modulate the autophagic process, which can serve as a potential therapeutic in NSCLC.

Autophagy is a multi-step self-eating process where every single phase displays a unique set of characteristics while maintaining cellular homeostasis. On the whole, the process of autophagy can be broadly classified into early and late phases, which signify the formation of uncommon complexes and the importance of autophagy initiation. Focusing on its initial insights, there are certain principal regulators like ULK1, ATG4B, and VPS34 that are in charge of Class III PI3K complex formation. Being a critical player in a cell’s biological phenomena, the complex is also known to modulate the later stages of internal processes. Of these, ULK1 and ULK2 are serine/threonine kinases and, hence, can be important targets for uncontrolled cell survival. Intriguingly, previous research has reported the efficacy of MRT67307 and MRT68921 towards ULK1 and ULK2, which repress autophagy upon mTOR suppression and persuade apoptotic behavior in cancer cells [226]. As discussed earlier about the role of Beclin-1 as a pro-autophagic molecule, anti-apoptotic proteins like Bcl-xL and Bcl-2 impede its activity by binding to its BH3 domain. Also, the effects of wortmannin and 3-MA have been analyzed to modulate Beclin-1’s interaction with VPS34, preventing autophagy [227]. These established references suggest the potential of both ULK1/2 and Beclin-1 inhibitors to suppress the autophagic pathway and improve apoptosis in cells bearing tumor antigens. The maturation of growing double-membrane structures into an autophagosome is assisted by cysteine proteases like ATG4B, together with ATG7 and ATG10. In vitro and in vivo studies suggest the efficacy of NSC185058 against the ATG4B enzyme in promoting anti-tumor activity [228]. In addition, other studies demonstrate that the increase in CD8+ infiltrating cells prevents autophagy by mutating a copy of the ATG7 protein [229]. However, while the result towards autophagy inhibition seems promising, more research is still being conducted to improve lead medications to reduce tumor survival in lung cancer patients. The clinical success of CQ as an autophagy inhibitor shows the therapeutic potential of autophagy inhibition [230], but it also emphasizes the urgent need for the expansion of new compounds in cancer therapy. Emphasizing the need for an hour for the development of antagonists to target later autophagic events can serve as a milestone towards anti-tumor therapy. In a recent study by Lu Zhang and his group, the discovery of a novel autophagy inhibitor, CUR5g, has displayed a potent therapeutic effect in NSCLC. Without an immediate impact on lysosome activity, it is believed to restrict autophagy by preventing autophagosome-lysosome fusion by neutralizing the acidic environment of lysosomes [231]. Other organic substances have been found in recent years to control autophagy and cause cancer cell death. In the treatment of several tumor types, fixetine inhibits the PI3K/Akt/mTOR pathway in human NSCLC cells, which can control autophagy [232]. Psoralen, a chemical compound obtained from the polymorph of *Angelica sinensis*, has been shown to control autophagy and apoptosis. By phosphorylating mTOR, angelicin promotes autophagic proteins like ATG3, 5, 7, and 12 [233]. In order to treat cancer cells, natural compounds may either encourage or prevent autophagy. These components can also be used to target cancer-progressing autophagy regulators like Beclin-1, mTOR, NFkB, Erk, Akt, and ROS. As a result, by controlling autophagy, natural chemicals may have huge therapeutic implications in cancer treatment [234].

## 10. Potential Limitations and Challenges of Targeting Autophagy in Lung Cancer Treatment

As discussed in the paper regarding the role of autophagy in promoting inflammatory responses, its long-term effect has been shown to remodel cancer treatment and drive host immunosuppression. Being an essential homeostatic process, it establishes a pro-tumor effect in cancer-infected cells. There are several studies that highlight the identification of pharmacological inhibitors against autophagic proteins. CQ and HCQ are two lysomotropic agents that have been used to treat malarial infections and rheumatological disorders. The use of these to overcome existing cancer during clinical assessment has shown improved outcomes. Additionally, the discovery of other specific inhibitors has shown to have a potent effect on almost all stages of autophagy. Also, screening for the best possible target has revealed immune infiltration at tumor sites and modulations in the autophagy process. Despite their functional relevance and therapeutic implications, there are still challenges in successful autophagic inhibition [235,236,237,238]. (1) Upon exposure of these molecules in cancer patients, the inhibitor showed molecular perturbations that increased infection severity. Similarly, in lung cancer, targeting autophagy may reflect molecular dynamics and interfere with the other signaling events for further tumor development. Considering the above fact, it therefore becomes important to quantify cell-specific markers for cellular perturbations. (2) According to the available literature, autophagy inhibition in lung cancer cells may lead to the overexpression of an important anti-tumor protein that complicates disease severity. (3) It can also lead to a change in circulating metabolites that have had an impact on repurposing inhibition kinetics. (4) Being a conserved cellular defense, the inhibitory effects on autophagic flux are desirable for a long-lasting effect that may not be reciprocated to favor defense in healthy cells. (5) Also, it may reverse the stimulatory effect of those accessory proteins that mediate autophagy and may modulate immune mechanisms towards immunosuppression. In order to overcome the above-underlined potential limitations, genetic models could be great for addressing the inhibitor’s pharmacological activity. Amalgamating autophagic deletion studies could help provide insights into cancer progression due to adaptive immune perturbations that can be rescued by tuning the reaction kinetics and inhibitory flux. However, RNA technology and cassettes can be used to specifically inhibit pathways that may have little or no effect on non-specific targets. To advance this level of understanding, modeling a mathematical model while mimicking intricate biological conditions would provide a fundamental understanding that may be utilized to fine-tune the autophagic process. Moreover, interpreting the systems biological analysis at the host cellular level can facilitate individualized medicine for lung cancer patients.

## 11. Real World Examples or Case Studies to Illustrate the Practical Application of Systems Biology in Cancer Research

To date, the practical implications of systems biology in cancer immunotherapy are not very evident. This is due to the fact that studies at present are more inclined to gain insights into the cellular and molecular aspects of NSCLC. With its advent around 2000, systems biology has paved the way to resolve biological complexity, signaling networks, and perturbed cellular events. Studies are being conducted to elucidate the network integrity of established infections, like cancer, which has provided various intricate information regarding a complex biological system [239,240,241]. Recalling the contributions of the systems biology approach, it provides knowledge about the over-expressed protein candidate, immune-molecular dynamics, and the factors that potentiate metastasis in nearby tissues. Collecting and integrating this data would help provide information about actionable nodes that regulate the pathway of cancer signaling and autophagic flux during infection. Altogether, the above-established findings will help us to explore minute details, which can be further utilized to arrive at the idea of achieving systems immuno-oncotherapeutics.

## 12. Concluding Remarks

NSCLC is a leading cause of cancer-related death worldwide, but its treatment has remained elusive for ages. Investigating the route of origin and the molecular theories behind NSCLC will help improve disease prognosis and outcome [242]. Autophagy is a physiological cell death process essential for maintaining cellular balance and homeostasis to sustain cell metabolism in healthy cells. To understand the influence that the IL6-IL-17-IL23 signaling axis has on autophagy, facilitating chronic inflammation, is therefore needed to explore the intricacies between innate immunological defense mechanisms. With a better understanding of the function and mechanics of autophagy, the issues with which we are faced today will be undoubtedly more complicated than initially believed. Considering the influence autophagy has on cancer therapy, the crucial question that arises is whether targeting the process would enhance or mitigate the outcome of chemotherapeutic drugs. Additionally, the role of inflammasomes on the macroautophagy pathway and its pro-tumor effect have inclined researchers more towards understanding and dissecting the crosstalk that promotes tumor growth. However, the assembly of inflammasome components produces cytokines that induce a signaling cascade and aggravate inflammatory responses [141]. Still, understanding specific cytokines is inadequate due to the dynamic nature of cancer-associated genes and their characteristics. Therefore, at this stage, it is required to employ different approaches to understand the biological systems and their response to environmental changes. To understand the intricacies of biological systems, the field of systems biology evolved as a systematic approach that integrates quantitative data, mathematical modeling, and computational biology methods. Recently, systems biology, due to its broader perspective, has been adopted as a holistic approach to compensate for the unresolved issues underlying cancer inflammation. It also offers a means by which the knowledge can be translated to lung cancer patients [243].

## Abbreviations

3-MA	3-methyladenine
AMPK	AMP-activated protein kinase
APC	Antigen-presenting cell
ASC	Adaptor apoptosis-associated speck-like protein
*ATG*	autophagy-related genes
Bif1	Bax-interacting factor-1
BMP	Bone morphogenetic protein
CAF	Cancer-associated fibroblast
CARD	Caspase activation and recruitment domain
ChIP	Chromatin immunoprecipitation
CHMP2A	Chromatin-modifying protein/charged multivesicular body protein 2A
CMA	Chaperone-mediated autophagy
CQ	Chloroquine
DC	Dendritic cell
EC	Endothelial cell
EGF	Epidermal growth factor
EGFR	Epidermal growth factor receptor
EMT	Epithelial mesenchymal transition
ESCRT	Endosomal complexes required for transport
FIP200	the focal adhesion kinase family interacting protein of 200 kD
GABARAP	GABA Type A Receptor-Associated Protein
GATE-16	Golgi-associated ATPase enhancer of 16 kDa
GRN	Gene regulatory network
GSDMD	Gasdermin D
HCC	Hepatocellular carcinoma
*HIF-1α*	hypoxia-inducible factor-1α
HIV	Human immune deficiency virus
HMGB1	High mobility group box 1
HQ	Hydroxychloroquine
HSC70	Heat shock cognate 71 KDa protein
IBD	Inflammatory bowel disease
IFN-γ	Interferon-gamma
IL	Interleukin
JNK	Jun N-terminal kinase
LAMP2A	Lysosomal membrane protein
LC	Lung Cancer
LC3	Microtubule-associated protein 1A/1B-light chain 3
LPS	Lipopolysaccharide
MAPK	Mitogen-activated protein kinase
MAPK	Mitogen-activated protein kinase
MCSF	Macrophage colony stimulating factor
MDSC	Myeloid-derived suppressor cells
MKK	Mitogen-activated protein kinase kinase
MMP	Matrix metalloproteinase
mTOR	mammalian target of rapamycin
NACHT	a central nucleotide binding and oligomerization
NK	Natural killer cell
NLRP3	NLR family protein containing a pyrin domain 3
NSCLC	Non-Small Cell Lung Cancer
PDGF-b	Plate-derived growth factor
PE	Phosphatidyl ethanol amine
PI3K	Phosphoinositide-3-kinase
PI3KC3	Phosphatidylinositol 3-phosphate kinase class III
PI3P	Phosphatidylinositol-3-phosphate
PIP2	Phosphatidylinositol 4,5-bisphosphate
PIP3	Phosphatidylinositol-3,4,5-trisphosphate
PPI	Protein-protein interaction
pTEN	a phosphatase and tensin homolog
RORγt	Rorc RAR-related orphan receptor gamma
ROS	Reactive oxygen species
SCLC	Small Cell Lung Cancer
scRNA	Single-cell RNA sequencing
SNARE	Soluble N-ethylmaleimide-sensitive-factor attachment protein receptor
STAT	Signal transducer and activator of transcription
TAM	Tumour-associated macrophages
TAN	Tumor-associated neutrophil
TB	Tuberculosis
TC	Tumor Cell
TF	Transcription factor
TGF-β	Transforming growth factor-b
Th	T helper
TME	Tumor microenvironment
TNF-a	Tumor necrosis factor-a
Treg	T-regulatory cell
TSC	Tuberous sclerosis
ULK1	unc-51-like kinase 1
UVRAG	UV radiation resistance-associated gene
VEGF	Vascular endothelial growth factor
VPS	Vacuolar protein sorting
